# Evaluating the nonlinear effects of sleep duration on biological aging across phenotypic, genomic, and epigenomic data

**DOI:** 10.18632/aging.206306

**Published:** 2025-08-25

**Authors:** Xueyao Wu, Xunying Zhao, Aaron Ge, Zhitong Han, Can Hou, Yu Hao, Jinyu Xiao, Mengyu Fan, Stephen Burgess, Jiayuan Li, Xia Jiang

**Affiliations:** 1 Department of Epidemiology and Health Statistics, West China School of Public Health and West China Fourth Hospital, Sichuan University, Chengdu, China; 2 University of Maryland School of Medicine, Baltimore, MD 21201, USA; 3 School of Life Sciences, Sichuan University, Chengdu, China; 4 West China Biomedical Big Data Center, West China Hospital, Sichuan University, Chengdu, China; 5 MRC Biostatistics Unit, University of Cambridge, Cambridge CB2 0SR, United Kingdom; 6 British Heart Foundation Cardiovascular Epidemiology Unit, Department of Public Health and Primary Care, University of Cambridge, Cambridge CB2 0BD, United Kingdom; 7 Department of Clinical Neuroscience, Center for Molecular Medicine, Karolinska Institutet, Solna, Stockholm, Sweden; 8 Department of Nutrition and Food Hygiene, West China School of Public Health and West China Fourth Hospital, Sichuan University, Chengdu, China

**Keywords:** sleep duration, biological age, nonlinear relationship, causal inference, heritability enrichment

## Abstract

Short and long sleep durations have been inconsistently linked to aging and health outcomes, potentially due to underexplored nonlinear associations. Using phenotypic and genomic data from the UK Biobank (n=442,664), we applied multivariable linear regression, restricted cubic splines, and Mendelian randomization (MR) to analyze nonlinear relationships between self-reported sleep duration and biomarkers of accelerated aging: PhenoAge acceleration (PhenoAgeAccel), BioAge acceleration (BioAgeAccel), and leukocyte telomere length (LTL). Functional annotation analyses were performed to assess potential shared biological pathways using epigenomic profiles. Observational analyses supported U-shaped phenotypic associations between sleep duration and PhenoAgeAccel/BioAgeAccel, with optimal sleep around 7 h/d. For LTL, linear models suggested a U-shape, while spline models indicated an inverted reverse J-pattern. MR analyses corroborated the deleterious impacts of insufficient, but not excessive, sleep, by revealing a threshold nonlinear relationship between increasing genetically-predicted sleep duration up to 7 h/d and lower PhenoAgeAccel/BioAgeAccel, and a linear relationship with longer LTL. Cell-type enrichment analyses connected short sleep to BioAgeAccel/LTL through pathways related to muscle maintenance and immune function. These findings suggest that extending sleep may mitigate accelerated aging, though further research is needed to clarify the underlying biological mechanisms and whether excessive sleep also contributes causally to biological aging.

## INTRODUCTION

Aging affects individuals both personally and societally [1]. Recognizing that relying solely on chronological age is insufficient to comprehend the internal physiological states nor the inter-individual variation in the rate and manner of aging [2], extensive efforts have been made to develop measures to capture the underlying aging processes at the biological level, also termed biological age [3]. These measures involve the use of individual or composite biomarkers that demonstrate associations with typically chronological age or mortality [4]. Of particular interest, clinical-parameter biological-age algorithms (i.e., PhenoAge [5] and BioAge [6]) and telomere length have been validated as among the most reliable predictors of aging outcomes and hold promise for routine application in large populations [7–9].

While the determinants of the rate of aging are intricate, it is possible to moderate the aging process through lifestyle interventions that influence metabolic processes. As one of these potentials, sleep assumes a significant role in daily life and functions as a restorative process facilitating both physical and mental recovery [10]. Accumulating evidence suggests that deviations from normal sleep duration may contribute to the premature development and progression of age-related conditions [11, 12]. However, the specific impact of insufficient or excessive sleep on accelerated biological aging remains uncertain [10, 13, 14]. Previous observational studies, often limited in scale and focused on linear effects, have yielded conflicting findings within specific populations [15–22]. Notably, existing studies have predominantly relied on binary categorizations of “short” and “long” sleep, rather than exploring the potentially diverse patterns across the entire duration continuum. A comprehensive investigation for potential non-linear dose-response relationships may elucidate the need for tailored interventions or recommendations across different ranges of sleep duration to extend a healthy lifespan and mitigate the risks of age-related health outcomes.

The lack of consensus in the existing evidence can be attributed, at least in part, to the vulnerability of conventional observational studies to residual confounding and reverse causality. While these limitations can be tackled by implementing an experimental study, it is unethical to conduct experiment of long-term sleep deprivation to confirm its causal effect in accelerating aging. In such case, Mendelian randomization (MR) provides an alternative means of uncovering the causal nature underlying a phenotypic association by using genetic variants (randomly distributed at conception) as proxies for life-long exposure risks [23]. Recent methodological advancements have introduced the doubly ranked method, a novel nonlinear MR approach that provides more robust stratum-specific estimates compared to conventional approaches [24]. Despite these developments, few nonlinear MR studies have been conducted in the realm of sleep duration and biological aging [25, 26].

**Figure 1:**
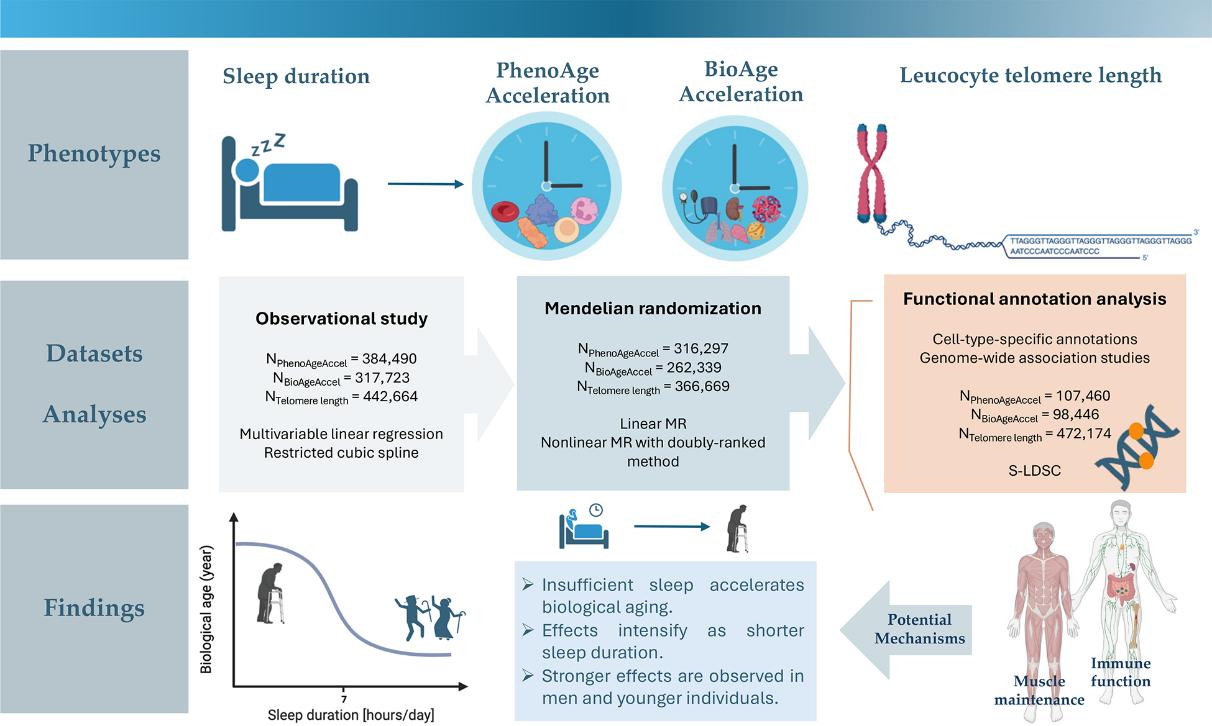
Graphical abstract.

Therefore, the present study aimed to provide a parametric visualization of the relationship between sleep duration and three key markers of biological aging status - PhenoAge acceleration, BioAge acceleration, and telomere length, utilizing extensive phenotypic and genotyping data from the UK Biobank. Multivariable regressions and restricted cubic spline analyses were first performed to examine phenotypic associations and whether phenotypic data support nonlinear effects. Linear and non-linear MR were then applied to delineate the shape of the causal relationships of genetically-predicted sleep duration with biological age measurements. Integrating genome-wide association study (GWAS) summary statistics and cell-type-specific annotations, functional annotation analyses were finally undertaken to interrogate potential genetic mechanisms relating sleep duration to the aging process. A graphical abstract is provided in Figure 1.

## MATERIALS AND METHODS

**Figure 2:**
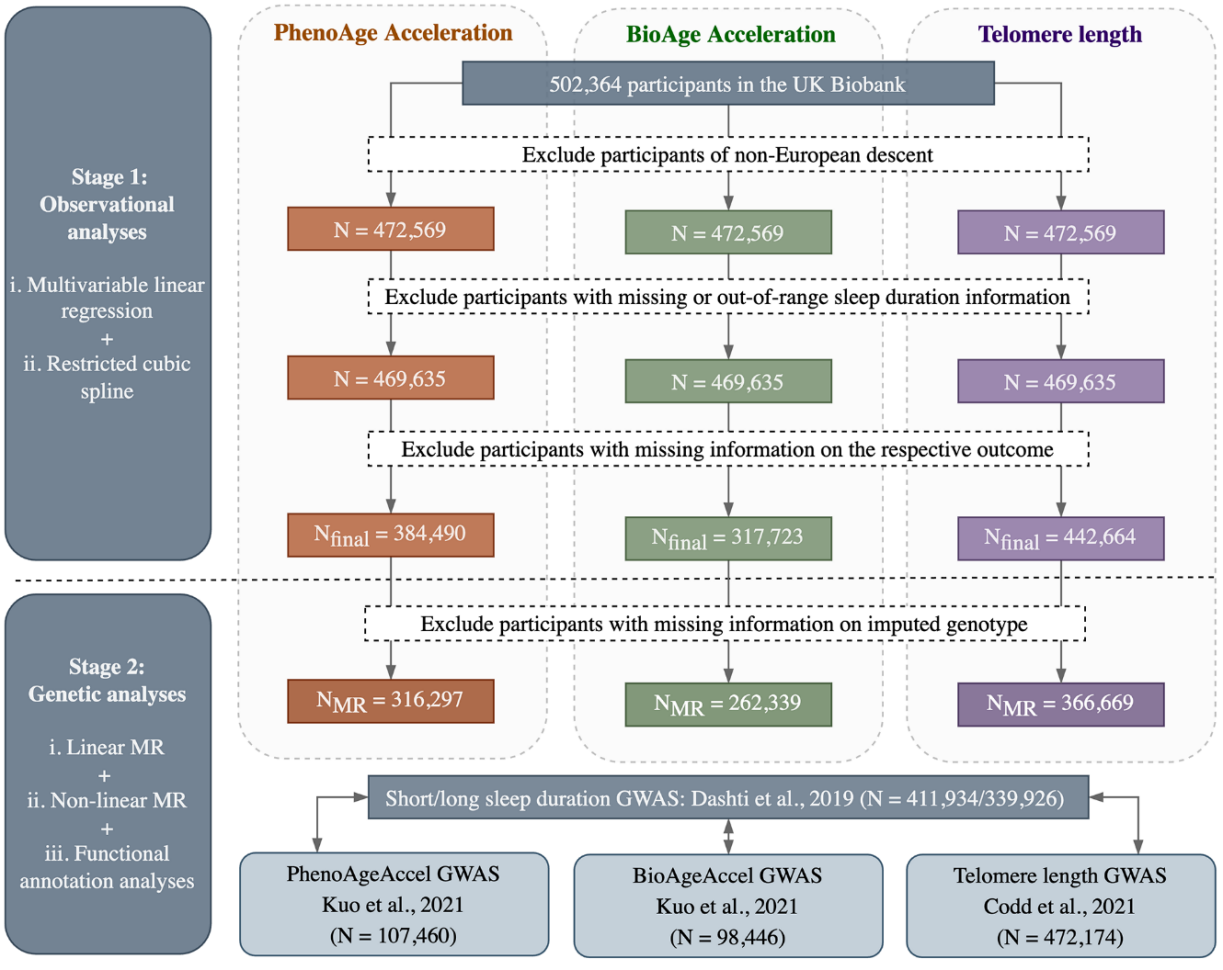
Flowchart of participant selection and overall study design.

The flowchart of participant selection and overall study design is outlined in Figure 2. In general, observational and MR analyses were conducted based on individual participant data from the UK Biobank, while functional annotation analyses were conducted using summary statistics from the hitherto largest GWAS of each phenotype. All analyses were performed in R (version 4.1.0) unless otherwise specified, with a two-sided *P*-value of 0.05 used as the threshold for statistical significance.

### Study participants

Participants were drawn from the UK Biobank, a large population-based prospective cohort study which recruited more than 500,000 individuals aged 40-69 years between 2006 and 2010. The study protocol is available online (www.ukbiobank.ac.uk/wp-content/uploads/2011/11/UK-Biobank-Protocol.pdf) and more details are published elsewhere [27]. At the initial visit, participants completed online questionnaire and physical measurements, and their biological samples were collected for genotyping and biochemistry tests. In our study, we used information on habitual sleep duration, biological traits for PhenoAge and BioAge construction, telomere length measurements, relevant confounding factors, and genetic variants. UK Biobank received ethical approval from the North West Multi-centre Research Ethics Committee and obtained written informed consent from all participants. Our study was performed under application number 99713.

### Self-reported habitual sleep duration

In the UK Biobank, participants’ habitual sleep duration was assessed at the baseline assessment via a standardized question: “About how many hours sleep do you get in every 24 hours? (please include naps)”, with responses in hour increment. Following the methods of previous studies [28, 29], participants with sleep durations below 2 hours or above 12 hours (extreme responses) were treated similarly to those who did not respond to the question. These participants along with those who responded with “Do not know” or “Prefer not to answer” were excluded from our analysis.

### PhenoAge, BioAge, and age accelerations

We computed two validated biological age predictors, PhenoAge [5] and BioAge [6], using two sets of nine clinical-based biomarkers involving a total of 12 blood chemistry and blood count traits as well as assessments of lung function and blood pressure. Details of the included biomarkers can be found in Supplementary Table 1. Both PhenoAge and BioAge were initially trained using data from the third National Health and Nutrition Examination Survey (NHANES III), but with different objectives. While PhenoAge was derived from an algorithm based on multivariate analysis of mortality hazards in the reference population to provide an estimate of the risk of death, BioAge was computed using an algorithm that involved a series of regressions of individual biomarkers on chronological age in the reference population, aiming to quantify the decline in overall system integrity. We applied the previously trained algorithms to the UK Biobank biomarker data to calculate PhenoAge and BioAge for each participant, using the R package “BioAge” [30]. To better quantify the differences between participants in biological aging, we utilized linear regression models to estimate the residual of the computed PhenoAge or BioAge after subtracting the effect of chronological age. These residuals were referred to as PhenoAge acceleration (PhenoAgeAccel) and BioAge acceleration (BioAgeAccel), respectively, and served as the primary outcomes in our analysis. Any observations with missing biomarkers values were excluded.

### Leukocyte telomere length

Telomeres are nucleoprotein complexes located at the ends of chromosomes that undergo shortening with each cell division. The length of telomere has been proposed as a biological measure of aging, reflecting the degree of cellular senescence and oxidative stress [31]. In this study, we focused on telomere length measured in leukocyte (LTL), a practical measure correlating well with telomere length across different tissues within individuals. In the UK Biobank, LTL measurements were ascertained on DNA from peripheral blood leukocytes collected at baseline assessment using a well-validated multiplex qPCR assay [32]. Measurements were reported as a ratio of telomere repeat copy number relative to that of a single copy gene (T/S ratios), which were then log-transformed to obtain a normal distribution (log_e_LTL). Multiple quality checks were applied to control and adjust for technical factors, as described elsewhere [32]. To allow direct comparison to other studies, we used z-standardized log_e_LTL as our primary outcome. Participants with missing LTL measurements were excluded.

### Genetic risk score of self-reported sleep duration

Genetic variants used in MR analyses were extracted genotypes from the UKB imputation dataset (n = 487,150 at the time of our study). Detailed information on genotyping, imputation, and quality control in the UK Biobank has been described previously [33]. We took 85 independent single-nucleotide polymorphisms (SNPs) robustly associated with continuous sleep duration (*P*-value < 5×10^−8^), obtained by applying PLINK’s clumping function [34] (parameters: -clump-r2 0.001 --clump-kb 1000) to results from the largest published GWAS of self-reported habitual sleep duration (n = 446,118) [35], as our genetic instruments. Details of the included SNPs are shown in Supplementary Table 2. Following SNP quality control [36], two palindromic SNPs (rs17732997 and rs4333549) were detected and subsequently excluded. To avoid one-sample bias towards confounded observational associations, we calculated an unweighted genetic risk score (GRS) by directly summing the number of sleep duration-increasing alleles across the 83 SNPs. Collectively, the unweighted GRS explained 6.22% of the genome-wide SNP-based heritability of sleep duration, as determined by comparing residual variance in linear regression models of sleep duration on GRS (*F*-statistic = 2,239).

### GWAS data sources

The largest published GWAS of self-reported habitual sleep duration also conducted separate GWAS for short (< 7 h/d; n = 106,192 cases) and long (> 8 h/d; n = 34,184 cases) sleep relative to 7–8 h/d sleep duration (n = 305,742 controls) [35]. We retrieved the full sets of summary statistics for the three sleep duration phenotypes for further functional annotation analyses. We also obtained the largest available GWAS of PhenoAgeAccel (n = 107,460) [37], BioAgeAccel (n = 98,446) [37], and LTL (n = 472,174) [32]. All original GWAS analyses were performed using imputed genotype data from the UK Biobank, involving only participants of European descent.

### Statistical analyses

### Observational analyses

Participants of European ancestry (to mirror the genetic analyses) with complete (and within range) exposure and outcome data were included. Separate multivariable linear regression models were employed to assess the relationships of continuous sleep duration with PhenoAgeAccel, BioAgeAccel, and LTL. In addition to the top five genetic principal components (PCs), factors previously described as associated with biological age measurements were included as covariates, including age, sex, educational qualifications (degree, no degree), body mass index, smoking history (current, former, or never), drinking history (current, former, or never), physical activity status (low, moderate, high), histories of cardiovascular disease (angina, heart attack, or stroke), hypertension, diabetes mellitus, and leucocyte count (for LTL as an outcome).

To assess the potential for nonlinear associations, we entered the levels of self-reported sleep duration as indicator variables, and obtained estimates comparing each of < 5 h/d, 5 h/d, 6 h/d, 8 h/d, 9 h/d, and > 9 h/d to our chosen reference category of 7 h/d. Restricted cubic spline regressions were further employed to model the non-linear relationships between continuous sleep duration and biological age measurements with four knots (located at the 5th, 35th, 65th, and 95th percentiles) after adjustments.

Considering the previously observed sex differences in biological aging [32, 38] and the age-dependent variations in sleep duration [39], all analyses were additionally stratified by sex and age (using the median age of 58 years as the cutoff point).

### Mendelian randomization analyses

Participants of European ancestry (to avoid population stratification) with complete data on sleep duration, aging outcomes, and genotypes were further included in the MR analyses. We first applied a two-stage method to estimate the average causal effect of sleep duration on the outcomes. This conventional MR approach tests for the presence of a causal relationship, yielding effect estimates under a linear modeling framework. In the first-stage analysis, we regressed the exposure (sleep duration) on the unweighted GRS. In the second stage, we regressed the outcomes on the fitted values of the exposure from the first stage. The regression models in both stages were adjusted with age at baseline, sex, age-squared, age-by-sex, age-squared-by-sex, the top five PCs of ancestry, and genotyping array.

While linear MR was conducted in the overall population to estimate averaged causal effects, nonlinear MR involves generating strata within the study sample and undertaking MR analyses within each stratum [40]. We applied the fractional polynomial method to examine nonlinearity and used the recently developed doubly-ranked stratification approach [24] to construct five equal-sized strata of the population. Compared to the conventional residual-based method [40], the doubly-ranked approach offers increased reliability when the genetic effects on the exposure vary across the population, or when the exposure measurement is coarsened (e.g., self-reported sleep duration measured in hourly units) [24]. We first stratified all participants into preliminary strata (each containing 50 participants) according to the levels of the GRS, and then stratified them into final strata based on the levels of observed sleep duration within each pre-stratum. This ensures that the constructed strata are uncorrelated with the GRS, while also guaranteeing that the average exposure level increases monotonically across the strata.

For each final stratum, we calculated a linear MR estimate of the causal effect of increasing sleep duration using the two-stage method described above. To stabilize estimates, we performed a bootstrap averaging approach. We randomly removed a small number of participants (n = 12) from the analysis and performed the doubly-ranked approach using this dataset. We repeated this procedure 100 times and then combined the estimates using Rubin’s rules. To evaluate the presence of a trend in the stratum-specific estimates, we performed a meta-regression of these estimates on the mean value of observed sleep duration in each stratum [40, 41], considering a *P*-value < 0.05 as evidence supporting nonlinearity. Sex- and age-stratified MR analyses were also performed.

Two sensitivity analyses were performed. First, we incorporated all additional confounding factors included in the multivariable regression models into the two-stage MR analyses to address potential residual confounding. Second, to assess the MR assumption that genetic variants influence biological aging solely through sleep duration, we recalculated the unweighted GRS after disregarding six potential pleiotropic SNPs that demonstrated associations (*P*-value < 5×10^−8^) with phenotypes other than sleep duration, as indicated by the GWAS catalog [42], and repeated MR analyses. The potential pleiotropic SNPs and their corresponding related phenotypes are shown in Supplementary Table 2. Given the methods for exploring horizontal pleiotropy in one-sample MR can be underpowered in each stratum, we compared the effect estimates derived from sensitivity analyses with those obtained from the primary analyses to assess the consistency of patterns. All MR analyses were performed using the R packages “ivreg” and “SUMnlmr”.

### Functional annotation analyses

To elucidate the potential shared biological mechanisms underlying the observed relationships between sleep duration and biological aging, we conducted functional annotation analyses by partitioning the SNP-heritability of each phenotype based on cell-type-specific annotations and examining their clustering patterns. A total of 396 annotations from the Roadmap Epigenomics project encompassing six histone marks (DNase, H3K27ac, H3K36me3, H3K4me1, H3K4me3, and H3K9ac) across 88 primary cell types or tissues [43] were utilized. These annotations were further categorized into nine broad groups, including adipose, central nervous system (CNS), digestive system, cardiovascular, musculoskeletal and connective tissue, immune and blood, liver, pancreas, and other. For each phenotype, annotation-specific enrichment values were calculated using stratified-LDSC [44], which were then transformed into a color scale and visualized through hierarchical clustering. FDR-adjusted *P*-value was applied based on the specific numbers of comparisons made in each analysis.

### Data availability

The data underlying the results presented in the study are available to researchers upon application to UK Biobank (https://www.ukbiobank.ac.uk/).

### Consent to participate

The participants in the UK Biobank study provided written informed consent for their data to be used in health-related research. As this study analyzes aggregated GWAS summary statistics that do not contain any personally identifiable information, no additional consent for publication is required.

### Consent to publish

The participants in the UK Biobank study provided written informed consent for their data to be used in publications.

## RESULTS

### Study population

Table 1 summarizes the baseline characteristics of study participants included in the observational analyses of PhenoAgeAccel (n = 384,490), BioAgeAccel (n = 317,723), and LTL (n = 442,664). The mean age of participants ranged from 56.6 to 56.8 years, and 53.7% to 54.2% of participants were women. More detailed characteristics of the three partially overlapping groups of participants according to self-reported sleep duration are presented in Supplementary Tables 3–5.

**Table 1:** Baseline characteristics of UK Biobank participants included in observational analyses.

**Characteristics**	**Analysis of PhenoAgeAccel**	**Analysis of BioAgeAccel**	**Analysis of LTL**
No. of participants	384490	317723	442664
Sleep duration, h/d	7.16 (1.08)	7.16 (1.06)	7.16 (1.08)
Age at recruitment, y	56.8 (8.02)	56.6 (8.04)	56.8 (8.03)
Sex (women), n (%)	206642 (53.7%)	171835 (54.1%)	239936 (54.2%)
Education			
Degree, n (%)	315151 (82.7%)	263167 (83.5%)	363075 (82.7%)
No degree, n (%)	66008 (17.3%)	51954 (16.5%)	75785 (17.3%)
Body mass index, kg/m^2^	27.4 (4.75)	27.3 (4.67)	27.4 (4.76)
Smoking status			
Never, n (%)	206510 (53.9%)	172605 (54.5%)	237954 (53.9%)
Previous, n (%)	136638 (35.7%)	112269 (35.5%)	157078 (35.6%)
Current, n (%)	40062 (10.5%)	31820 (10.0%)	46137 (10.5%)
Drinking status			
Never, n (%)	12340 (3.21%)	10002 (3.15%)	14243 (3.22%)
Previous, n (%)	13271 (3.45%)	10414 (3.28%)	15299 (3.46%)
Current, n (%)	358575 (93.3%)	297067 (93.6%)	412778 (93.3%)
IPAQ activity group			
High, n (%)	126921 (40.5%)	106018 (40.9%)	146179 (40.5%)
Moderate, n (%)	127813 (40.8%)	105833 (40.8%)	147130 (40.8%)
Low, n (%)	58433 (18.7%)	47654 (18.4%)	67260 (18.7%)
Leukocyte count, 10^9 cells/Litre	/	/	6.90 (2.04)
Major diseases			
Cardiovascular disease, n (%)	22051 (5.74%)	16096 (5.07%)	25440 (5.76%)
Hypertension, n (%)	103404 (26.9%)	82698 (26.1%)	118845 (26.9%)
Diabetes mellitus, n (%)	18386 (4.79%)	14409 (4.54%)	21267 (4.81%)
Components of biological ages			
Lymphocyte (%)	28.7 (7.34)	/	/
Mean cell volume (fL)	82.9 (5.25)	/	/
Serum glucose (mmol/L)	5.11 (1.21)	/	/
Red cell distribution width (%)	13.5 (0.95)	/	/
White blood cell count (1000 cells/uL)	6.89 (1.93)	/	/
Albumin (g/L)	45.2 (2.61)	45.3 (2.59)	/
Creatinine (umol/L)	72.2 (16.2)	72.2 (17.1)	/
C-reactive protein (mg/dL)	0.26 (0.44)	0.25 (0.41)	/
Alkaline phosphatase (U/L)	83.5 (26.1)	83.0 (25.9)	/
FEV_1_ (L)	/	2.85 (0.80)	/
SBP (mm Hg)	/	138 (18.5)	/
Total Cholesterol (mg/dL)	/	221 (43.9)	/
Glycated hemoglobin (%)	/	3.58 (0.63)	/
Blood urea nitrogen (mg/dL)	/	15.2 (3.82)	/
Biological ages, y			
PhenoAge	50.8 (9.42)	/	/
PhenoAge acceleration	0.00 (4.66)	/	/
BioAge	/	53.9 (8.67)	/
BioAge acceleration	/	0.00 (3.31)	/
Z-standardized leucocyte telomere length	/	/	-0.01 (0.99)

Characteristics were presented as mean values (standard deviation) for continuous variables and n (%) for categorical variables.

PhenoAgeAccel, PhenoAge acceleration; BioAgeAccel, BioAge acceleration; LTL, leucocyte telomere length.

### Linear and nonlinear phenotypic associations

**Figure 3:**
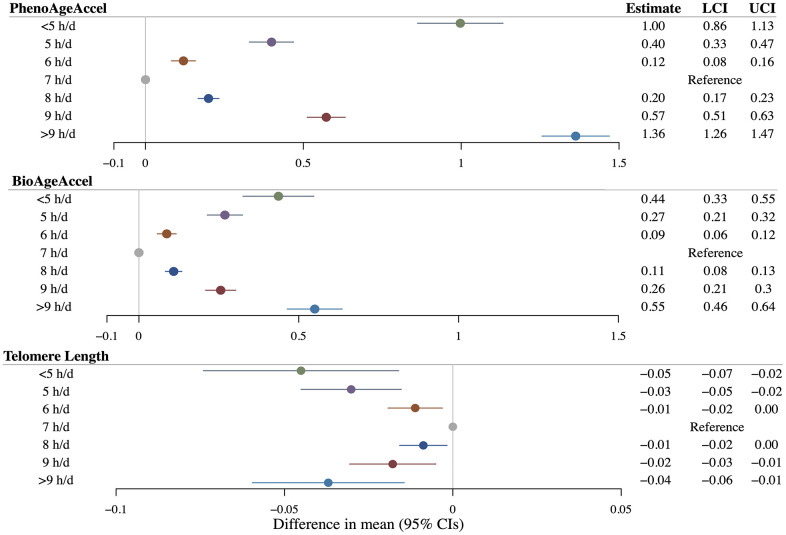
**Results of multivariable linear regressions of PhenoAge acceleration (PhenoAgeAccel), BioAge acceleration (BioAgeAccel), and leukocyte telomere length on sleep duration.** Circles denote point estimates. Error bars denote 95% confidence intervals. Regression models adjusted for: age at baseline, sex, the top five genetic principal components, educational qualifications, body mass index, smoking history, drinking history, physical activity status, leucocyte count (for telomere length as an outcome), and histories of cardiovascular diseases, hypertension, and diabetes mellitus.

Linear observational analyses revealed significantly positive associations between continuous sleep duration with both PhenoAgeAccel (*β* = 0.08 years per additional hour of sleep duration, 95%CI = 0.07 to 0.09) and BioAgeAccel (*β* = 0.02 years per additional hour of sleep duration, 95%CI = 0.01 to 0.03), but not with LTL (*β* = 0.0003 SD change per additional hour of sleep duration, 95%CI = –0.0024 to 0.0029) (Supplementary Table 6). Compared to participants reporting 7 hours of sleep per day, those reporting shorter (< 7 h/d) or longer (> 7 h/d) sleep durations showed significantly higher PhenoAgeAccel and BioAgeAccel, as well as significantly shorter LTL (Figure 3). Restricted cubic spline regressions also demonstrated U-shaped nonlinear associations of sleep duration with PhenoAgeAccel and BioAgeAccel, while suggested an inverted reverse J-shaped association of sleep duration with LTL (Supplementary Figure 1). All spline analyses yielded a *P*-value for nonlinearity below 0.001.

Sex- and age-stratified observational analyses demonstrated similar patterns of nonlinear associations, despite slightly larger statistical uncertainties (Supplementary Table 6 and Supplementary Figure 1). Notably, the magnitude of associations was consistently higher in men than in women across all outcomes. Insufficient sleep exhibited seemingly stronger associations with both PhenoAgeAccel and BioAgeAccel in younger (< 58 years) compared to older participants (≥ 58 years).

### Linear and nonlinear mendelian randomization

**Figure 4:**
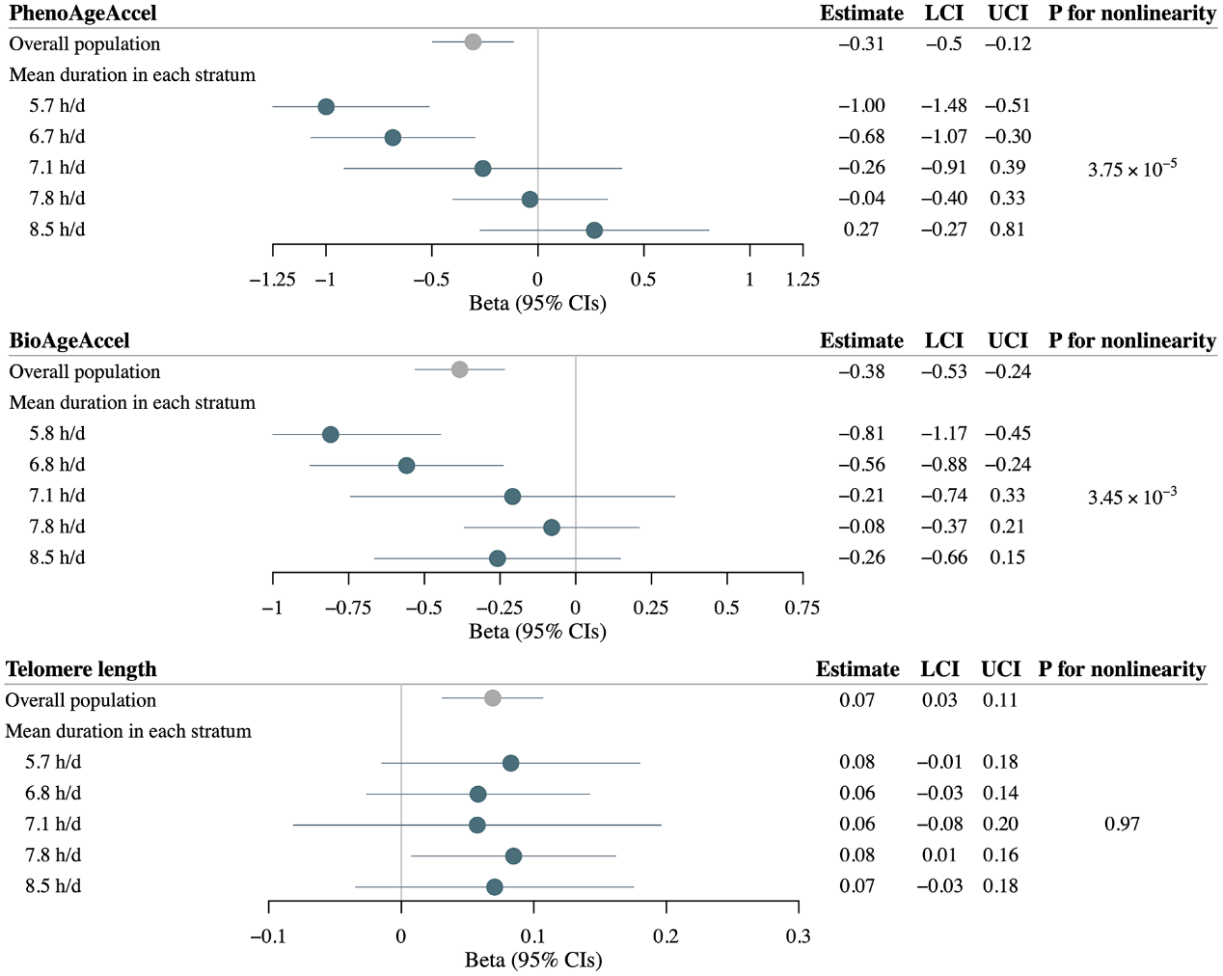
**Mendelian randomization estimates for PhenoAge acceleration (PhenoAgeAccel), BioAge acceleration (BioAgeAccel), and leukocyte telomere length in overall population and strata of population defined by residual sleep duration.** Grey circles denote overall estimates, and blue circles denote stratum estimates. Error bars denote 95% confidence intervals. Models adjusted for: age at baseline, sex, the top five genetic principal components, and genotyping array.

Utilizing 83 SNPs to instrument self-reported sleep duration, linear MR within the overall UK Biobank population found significant causal associations between genetically-predicted sleep duration and PhenoAgeAccel (*β* = –0.31 years per 1 hour increase in genetically-predicted sleep duration, 95%CI = –0.50 to –0.12), BioAgeAccel (*β* = –0.38 years per 1 hour increase in genetically-predicted sleep duration, 95%CI = –0.53 to –0.24), and LTL (*β* = 0.07 SD change per 1 hour increase in genetically-predicted sleep duration, 95%CI = 0.03 to 0.11) (Figure 4).

Stratifying the overall study sample into five strata, nonlinear MR demonstrated threshold nonlinear relationships for sleep duration with both PhenoAgeAccel and BioAgeAccel. We observed a strong inverse causal association of increasing genetically-predicted sleep duration with PhenoAgeAccel in the lowest strata (*β* = –1.00 years per 1 hour increase in genetically-predicted sleep duration, 95%CI = –1.48 to –0.51), an inverse but slightly weaker association in the second lowest stratum (*β* = –0.68, 95%CI = –1.07 to –0.30), whereas no significant association in the other three strata with mean observed durations > 7.1 h/d. Similarly, the strongest inverse causal association of increasing genetically-predicted sleep duration with BioAgeAccel was found in the lowest stratum (*β* = –0.81 years per 1 hour increase in genetically-predicted sleep duration, 95%CI = –1.17 to –0.45), followed by the second lowest stratum (*β* = –0.56, 95%CI = –0.88 to –0.24). The trends in estimates were significant, as evidenced by the significant *P*-values for nonlinearity for both PhenoAgeAccel (*P* = 3.75×10^−5^) and BioAgeAccel (*P* = 3.45×10^−3^) (Figure 4).

Nonlinear MR demonstrated a significant positive causal association of increasing genetically-predicted sleep duration with LTL in the second highest stratum (*β* = 0.08 SD change per 1 hour increase in genetically-predicted sleep duration, 95%CI = 0.01 to 0.16). No significant association was observed in the other duration groups, likely due to lower power arising from reduced sample size, nor a significant trend across stratum estimates (*P* = 0.97) (Figure 4).

We found largely consistent results in the sex-stratified nonlinear MR analyses of each biological age measurement, although some confidence intervals included the null (particularly in analyses of LTL) due to reduced statistical power. Notably, compared to women-specific analyses, men-specific nonlinear MR yielded higher effect magnitudes of extremely insufficient sleep duration on PhenoAgeAccel (*β* = –0.92 vs. *β* = –0.90) and BioAgeAccel (*β* = –1.44 vs. *β* = –0.71) (Supplementary Tables 7, 8). Additionally, insufficient sleep duration demonstrated stronger effects in younger participants compared to older participants for PhenoAgeAccel (*β* = –0.86 vs. *β* = –0.65), BioAgeAccel (*β* = –0.96 vs. *β* = –0.72), and LTL (*β* = 0.15 vs. *β* = 0.12) (Supplementary Tables 9, 10).

Sensitivity analyses incorporating additional confounders or utilizing a reconstructed GRS largely recapitulated the primary results (Supplementary Tables 11, 12).

### Cell-type-specific heritability enrichments

Functional annotation analyses further revealed extensively distributed heritability enrichments for sleep duration phenotypes, predominantly in cell types of the CNS, as well as in other components related to blood/immune, pancreas, and musculoskeletal/connective tissues. Comparing the heritability enrichments between sleep duration and biological age measurements, we found that both short sleep (< 7 h/d) and LTL were significantly enriched in the fetal thymus annotation, a component of the blood/immune system. Additionally, short sleep showed significant enrichment in the fetal muscle leg annotation, while BioAgeAccel displayed enrichment in the foreskin fibroblast primary cells annotation, both representing components of the musculoskeletal/connective system. In contrast, no common significant enrichment was identified between long sleep (> 8 h/d) and any of the biological aging outcomes (Supplementary Figure 2).

## DISCUSSION

To the best of our knowledge, this is the first phenotypic and genetic analysis that systematically interrogates a nonlinear relationship between sleep duration and accelerated biological aging. Our observational study suggests U-shaped phenotypic associations of sleep duration with PhenoAgeAccel and BioAgeAccel and an inverted reverse J-shaped phenotypic association of sleep duration with LTL, with approximately 7 h/d as the optimal sleep duration. A comprehensive GRS-based MR study further confirmed the detrimental roles of insufficient sleep across all outcomes (in a dose-response manner for PhenoAgeAccel and BioAgeAccel), while finding no evidence to support deleterious impacts of excessive sleep on any of the biological aging measurements.

In utilizing various analytical approaches that designed specifically for linear relationships, we found that the direction of the effect estimates for sleep duration on biological age measurements largely differs when comparing the results of linear MR and multivariable linear regressions. Such disparity suggests that evaluating continuous sleep duration as a whole may lead to unreliable findings underscoring the need to consider potential nonlinear effects when studying its health impact.

PhenoAgeAccel and BioAgeAccel, recognized as the best validated measures for biological aging that could also be implemented with data available in the UK Biobank [6, 9, 45], were designed based on different assumptions to capture distinct aspects of the aging process. BioAge models biological age as the average physiology of an individual with the same age as the research subjects [6], while PhenoAge estimates biological age as the average physiology of an individual with the same risk of death as the research subjects [5]. Therefore, the U-shaped relationship between sleep duration and PhenoAgeAccel observed in our observational study aligns with previous findings that a sleep duration of 7 hours represents the optimal duration associated with lowest all-cause and other-cause mortality [46, 47]. However, leveraging robust genetic instruments, our nonlinear MR analyses failed to confirm a causative role of long sleep duration in increasing PhenoAgeAccel, indicating that the observed associations are likely due to residual confounding or/and reverse causation. Indeed, given long sleep itself can be a consequence of underlying health conditions or comorbidities, and that accelerated aging may cause fatigue or other symptoms that lead to increased sleep duration [39], the association between excessive sleep and accelerated biological aging may be more susceptible to these common limitations inherent in conventional observational studies. Notably, such disagreement between observational and MR findings was consistently observed for PhenoAgeAccel and BioAgeAccel, providing reassurance that the associations reflect genuine biological aging rather than artifacts specific to a particular method of biological age measurement.

Previous observational studies have demonstrated a detrimental role of both insufficient and excessive sleep in PhenoAgeAccel/BioAgeAccel. For example, a study involving 615 participants from the Baltimore Longitudinal Study of Aging reported significantly lower DNA methylation-based PhenoAge among individuals sleeping ≤ 6 h/d compared to those sleeping > 7 h/d [17]. Another study involving 29,309 participants from the Korean NHANES V-VI found significantly higher metabolism diagnostic parameters-based BioAgeAccel among individuals who sleep > 8 h/d compared to those sleeping between 6 to 8 h/d [15]. It is important to note that these previous studies, like many others investigating sleep duration, employed a block classification of “short” and “long” sleep duration that imposed a linear relationship between aging and more or less sleep around a chosen cut-off (e.g., 6, 7, or 8 h/d). This approach, coupled with relatively small sample sizes, limited their ability to detect the impact of different duration patterns or the magnitude of effects associated with more extreme sleep durations. Building upon previous findings, our work based on triangulated evidence supporting that an insufficient sleep duration (defined as < 7 h/d) may exert increasingly pronounced detrimental effects on accelerated biological aging as sleep insufficiency becomes more severe.

While PhenoAge and BioAge aim to capture integrated multi-system dysregulation during aging, LTL and its natural shortening primarily reflect cellular proliferative capacity [31]. To date, epidemiological evidence regarding the relationship of sleep duration and telomere length has remained highly inconclusive. As one of the pioneering studies in this field, an investigation involving 4,117 female participants from the Nurses’ Health Study reported a positive association between sleep duration (9 h/d compared to ≤ 6 h/d) and LTL [18]. However, a separate study with 245 women reported no significant association [19]. Similar mixed results have further been reported in samples of both men and women [13, 14], particularly regarding long sleep duration, which has been linked to both significantly longer [20] and shorter telomere length [21]. A previous exploratory study involving 154 participants indicated a U-shaped association between sleep duration and telomere length [22], which was recapitulated by our large-scale multivariable linear regressions. Nonetheless, stratified MR findings again indicate no causative role of excessive sleep in shortening LTL. Unlike the nonlinear relationships identified for the composite biological age measurements, the stratum estimates for LTL were more consistent across strata and centered around the population-averaged estimate, suggesting a linear effect. While such discrepancy may support the notion that these measurements capture different hallmarks of aging [48], additional investigations are warranted to corroborate our findings and provide deeper insights into the reasons behind the distinct associations observed.

Through the integration of genomics and epigenomics data, our study reveals genetic similarities between short sleep and BioAgeAccel in the musculoskeletal system, as well as between short sleep and LTL in the immune system, providing additional evidence for potential shared genetic mechanisms that contribute to these causal links. Sleep plays a crucial role in connective tissue repair and muscle growth [49], and inadequate sleep has been associated with increased risks of muscle mass reduction and functional decline commonly observed during aging [50]. Moreover, laboratory studies have demonstrated that both acute and chronic sleep loss can have an impact on a wide range of immune function [51]. The immune system is intricately connected to telomere shortening, as its proper functioning relies on the renewal and clonal expansion of T- and B-cell populations [52]. Emerging evidence suggests that immune dysfunctions also contribute to telomere and telomerase deficiency [52]. Taken together, our findings, along with the previous research, support the hypothesis that insufficient sleep adversely affects the musculoskeletal system and immune function, consequently accelerating biological aging. To gain a deeper understanding of these hypothesized mechanisms, future in-depth experimental studies are needed.

A notable strength of our study lies in the comprehensive interrogation of potential sex-heterogeneous effects of sleep duration on biological aging, revealing that short sleep duration accelerates aging more prominently in men than women. This finding aligns with prior research indicating that men are more susceptible to immunosenescence, inflammaging, and higher mortality risk at comparable frailty levels [38]. Importantly, while prior work reported sex-specific association between sleep duration and LTL in men [53], our findings extend this by demonstrating that sufficient sleep slows biological aging in both sexes. Similarly, our age-stratified analyses indicate that insufficient sleep accelerates biological aging in both younger (< 58 years) and older (≥58 years) adults, with a seemingly more pronounced effect observed in the younger group. This is consistent with evidence that younger individuals typically require longer sleep durations and demonstrate reduced resilience to acute sleep deprivation compared to their older counterparts [54]. Overall, these findings emphasize the importance of adequate sleep duration for healthy aging across the population, with particular attention to the needs of men and younger adults. Nevertheless, validation in larger, independent cohorts is necessary before these subgroup findings can inform clinical recommendations.

Several limitations need to be acknowledged. Our sample only comprises individuals of White European ethnic background, necessitating future investigations to ensure the generalizability of findings across diverse racial or ethnic groups. Additionally, the potential presence of a healthy volunteer bias within the UK Biobank may introduce a null bias, given the anticipated lower age accelerations relative to the general UK population. Sleep duration was assessed using a single self-administrated question in the UK Biobank. However, the potential for misclassification error is unlikely to influence our MR estimates as sleep duration is measured in 1-hour increments [55]. Future studies with objective sleep measures are essential to validate our findings. Our study specifically examined telomere length measurements in leukocytes, and further research is needed to determine how well these measurements reflect telomere length in other organ tissues. Finally, it is important to recognize the cross-sectional design of our observational study and approach the interpretation of causality between sleep duration and aging with caution. While MR offers some potential for causal inference, it relies on several assumptions [23] that we have tried to scrutinize, albeit residual uncertainty inevitably remains. For example, it remains possible that some genetic instruments exert direct effects on biological aging, which would violate the exclusion restriction assumption.

## CONCLUSIONS

Taken together, our study, utilizing extensive phenotypic, genomic, and epigenomic data, consistently demonstrates a significant association between insufficient sleep duration and increased acceleration of biological aging, as indicated by PhenoAgeAccel, BioAgeAccel, and LTL measurements. Further investigation is needed to determine if long sleep is a causal risk factor for accelerating biological aging. Findings suggest that interventions aimed at addressing insufficient sleep may serve as a pathway towards alleviating the burden of aging.

## Supplementary Material

Supplementary Figures

Supplementary Tables 1-5

Supplementary Table 6

Supplementary Table 7

Supplementary Table 8

Supplementary Table 9

Supplementary Table 10

Supplementary Table 11

Supplementary Table 12
