# Supplementary material for: Evaluating the nonlinear effects of sleep duration on biological aging across phenotypic, genomic, and epigenomic data
Source: Aging (Albany NY). 2025 Aug 25;17(8):2126–51. doi: 10.18632/aging.206306 (PMC12422793; doi:10.18632/aging.206306)
Supplement: Supplementary Figures [file aging-17-8-206306-s001.pdf]

SUPPLEMENTARY FIGURES

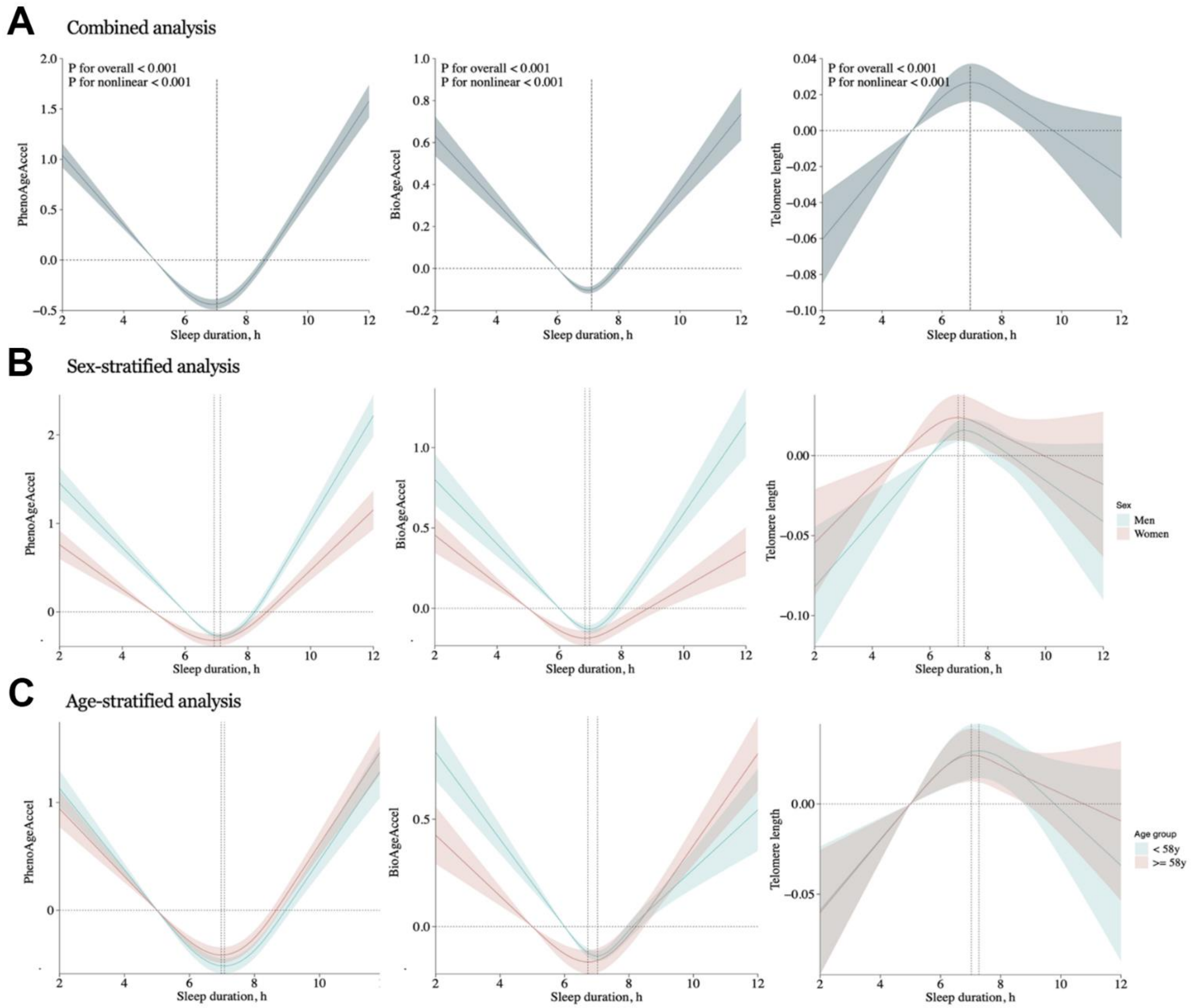

Supplementary Figure 1. Results of restricted cubic spline regressions of PhenoAge acceleration (PhenoAgeAccel), BioAge acceleration (BioAgeAccel), and leukocyte telomere length on sleep duration. Models adjusted for, where appropriate, age at baseline, sex, the top five genetic principal components, educational qualifications, body mass index, smoking history, drinking history, physical activity status, leucocyte count (for telomere length as an outcome), and histories of cardiovascular diseases, hypertension, and diabetes mellitus. (**A**) Combined analysis, (**B**) Sex-stratified analysis, (**C**) Age-combined analysis.

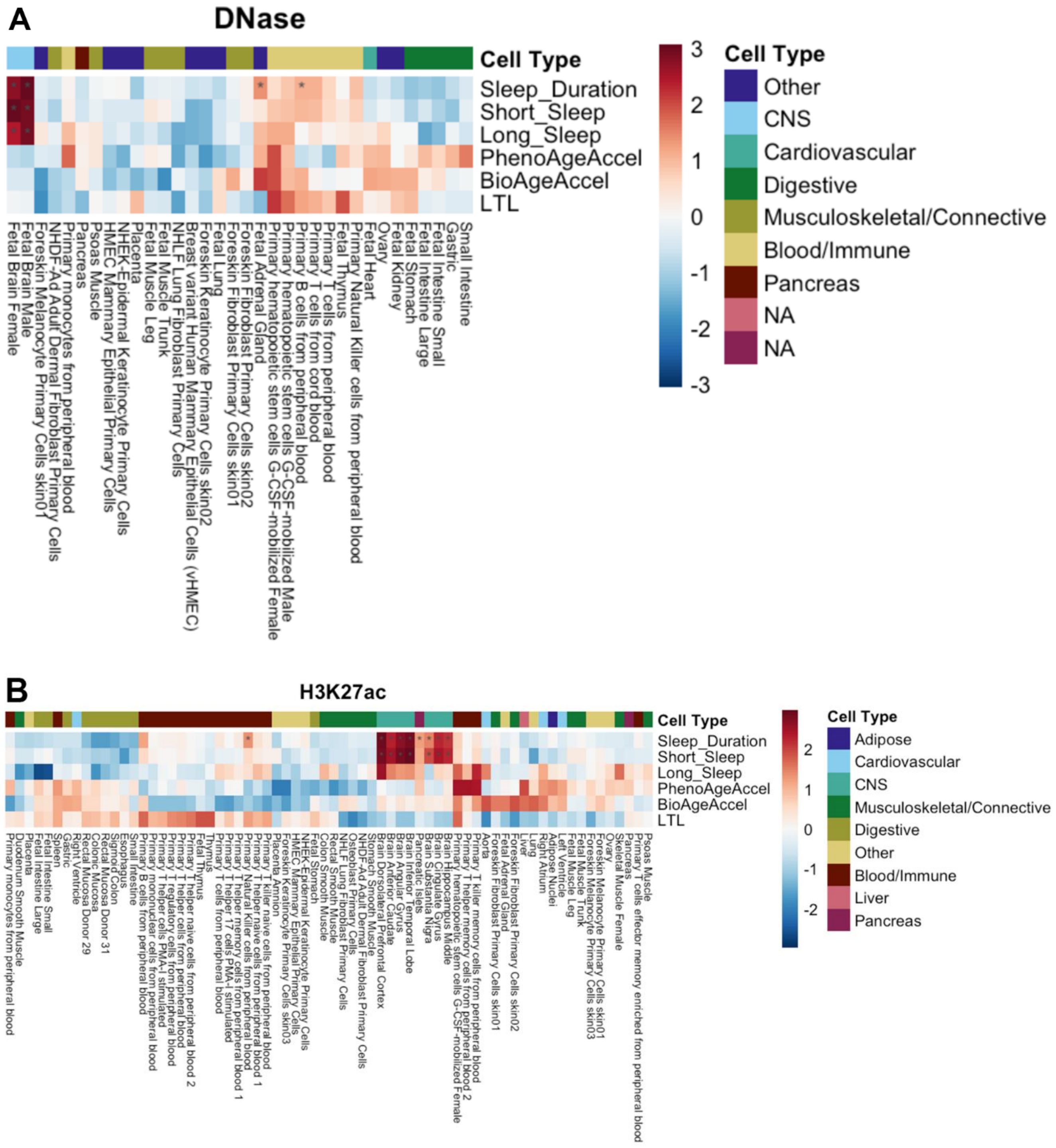
A
DNase
Cell Type
Sleep_Duration
Short_Sleep
Long_Sleep
PhenoAgeAccel
BioAgeAccel
LTL
Fetal Brain Female
Fetal Brain Male
Foreskin Melanocyte Primary Cells skin01
NHDF-Ad Adult Dermal Fibroblast Primary Cells
Primary monocytes from peripheral blood
Pancreas
Psoas Muscle
HMEC Mammary Epithelial Primary Cells
NHEK-Epidermal Keratinocyte Primary Cells
Placenta
Fetal Muscle Leg
Fetal Muscle Trunk
NHLF Lung Fibroblast Primary Cells
Breast variant Human Mammary Epithelial Cells (vHMEC)
Foreskin Keratinocyte Primary Cells skin02
Fetal Lung
Foreskin Fibroblast Primary Cells skin01
Foreskin Fibroblast Primary Cells skin02
Fetal Adrenal Gland
Primary hematopoietic stem cells G-CSF-mobilized Female
Primary hematopoietic stem cells G-CSF-mobilized Male
Primary B cells from peripheral blood
Primary T cells from cord blood
Primary T cells from peripheral blood
Fetal Thymus
Primary Natural Killer cells from peripheral blood
Fetal Heart
Ovary
Fetal Kidney
Fetal Stomach
Fetal Intestine Large
Fetal Intestine Small
Gastric
Small Intestine
3
2
1
0
-1
-2
-3
Cell Type
Other
CNS
Cardiovascular
Digestive
Musculoskeletal/Connective
Blood/Immune
Pancreas
NA
NA
B
H3K27ac
Cell Type
Sleep_Duration
Short_Sleep
Long_Sleep
PhenoAgeAccel
BioAgeAccel
LTL
2
1
0
-1
-2
Cell Type
Adipose
Cardiovascular
CNS
Musculoskeletal/Connective
Digestive
Other
Blood/Immune
Liver
Pancreas

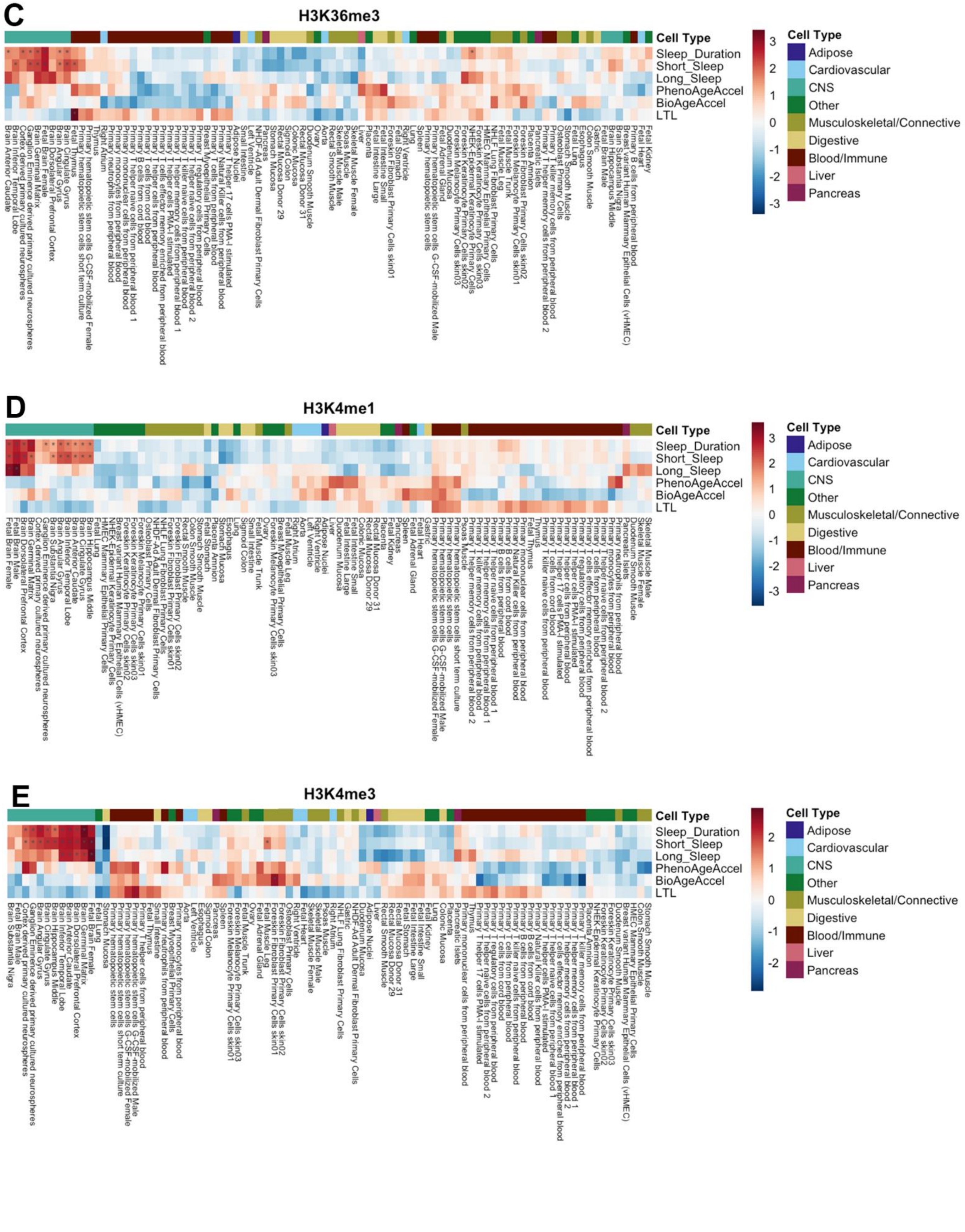
C
H3K36me3
D
H3K4me1
E
H3K4me3
Cell Type
Sleep_Duration
Short_Sleep
Long_Sleep
PhenoAgeAccel
BioAgeAccel
LTL
Adipose
Cardiovascular
CNS
Other
Musculoskeletal/Connective
Digestive
Blood/Immune
Liver
Pancreas

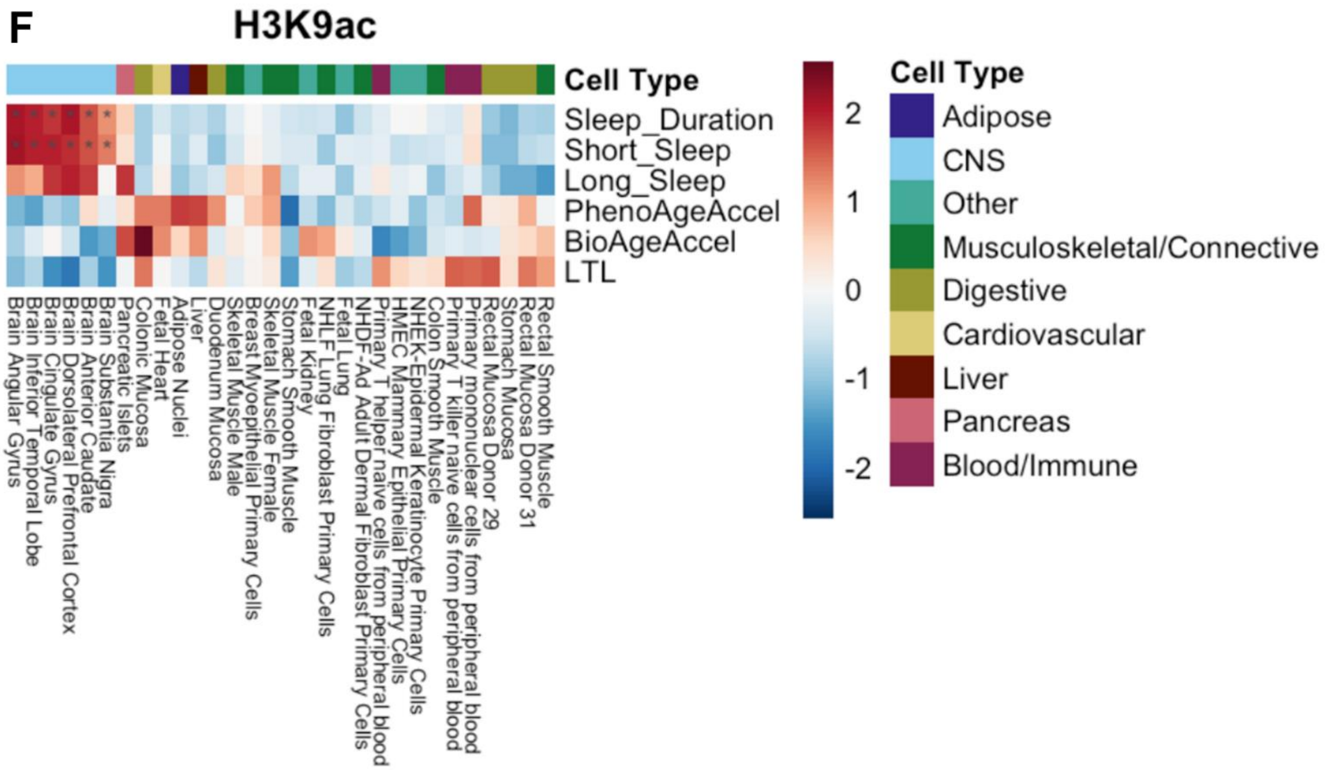

Supplementary Figure 2. Clustering of cell-type-specific annotation for sleep duration phenotypes and biological age measurements over histone marks. Each colored square reflects the z-score, scaled by traits. Red indicates enrichment, blue indicates depletion. Deeper color represents stronger magnitude of effects. Asterisks represent statistical significance withstanding correction (FDR-adjusted *P*-value < 0.05). (**A**). DNase, (**B**). H3K27ac, (**C**). H3K36me3, (**D**). H3K4me1, (**E**). H3K4me3, and (**F**). H3K9ac. PhenoAgeAccel: PhenoAge acceleration; BioAgeAccel: BioAge acceleration; LTL: leucocyte telomere length.
