# Supplementary material for: Evaluating the nonlinear effects of sleep duration on biological aging across phenotypic, genomic, and epigenomic data
Source: Aging (Albany NY). 2025 Aug 25;17(8):2126–51. doi: 10.18632/aging.206306 (PMC12422793; doi:10.18632/aging.206306)
Supplement: Supplementary Tables 1-5 [file aging-17-8-206306-s002.pdf]

SUPPLEMENTARY TABLES

Supplementary Table 1. Full names and field IDs of variables included in the current study.

Phenotypes	Labels in the current study	Full name in UK Biobank data dictionary	UK Biobank Field ID
Sleep duration	Sleep duration (h/d)	Sleep duration	1160
Components of biological ages	FEV_1 (L)	Forced expiratory volume in 1-second (FEV_1) (litres)	3063
	SBP (mm Hg)	Systolic blood pressure, automated reading (mm Hg)	4080
	Total Cholesterol (mg/dL)	Cholesterol (mmol/L)	30690
	Glycated hemoglobin (%)	Glycated haemoglobin (HbA1c) (mmol/mol)	30750
	Blood urea nitrogen (mg/dL)	Urea (mmol/L)	30670
	Lymphocyte (%)	Lymphocyte percentage (%)	30180
	Mean cell volume (fL)	Mean sphered cell volume (femtolitres)	30270
	Serum glucose (mmol/L)	Glucose (mmol/L)	30740
	Red cell distribution width (%)	Red blood cell (erythrocyte) distribution width (%)	30070
	White blood cell count (1000 cells/uL)	White blood cell (leukocyte) count (10^9 cells/Litre)	30000
	Albumin (g/L)	Albumin (g/L)	30600
	Creatinine (umol/L)	Creatinine (umol/L)	30700
	C-reactive protein (mg/dL)	C-reactive protein (mg/L)	30710
	Alkaline phosphatase (U/L)	Alkaline phosphatase (U/L)	30610
Leucocyte telomere length	Leucocyte telomere length	Z-standardized leucocyte telomere length values (LTL)	22192

Supplementary Table 2. Characteristics on genetic variants used for construction of polygenic risk score of self-reported sleep duration.

SNP	Chromosome	Base pair	Effect Allele	Reference Allele	BETA	EAF	Pleiotropic phenotypes
rs915416	1	34731984	C	G	0.0192587	0.289947	
rs269054	1	57864304	T	A	-0.0136431	0.577924	
rs61796569	1	66476437	C	T	-0.0154442	0.730417	
rs12567114	1	98527951	G	A	-0.0148307	0.724198	
rs62120041	2	9185564	T	C	0.0261113	0.933902	
rs374153	2	40382712	C	T	0.0176119	0.158085	
rs2717076	2	58061127	C	T	-0.0184107	0.372584	
rs75539574	2	58871658	A	C	-0.0362482	0.914208	
rs72804080	2	59358659	A	G	-0.0177935	0.850072	
rs7556815	2	114085785	G	A	-0.0407248	0.780856	
rs12611523	2	139195328	A	G	0.0126347	0.545244	
rs35662245	2	147583187	T	A	-0.0145968	0.661256	Schizophrenia
rs4538155	2	157040773	C	T	-0.0129753	0.352574	
rs11885663	2	166944004	C	T	-0.0162176	0.752191	
rs10173260	2	210377845	T	C	-0.0128368	0.393765	
rs112230981	3	55879269	A	G	0.0315275	0.94984	
rs17732997	3	70470834	C	G	0.0129345	0.569098	
rs7644809	3	107564459	T	C	0.0130621	0.421606	
rs13088093	3	135838598	T	G	-0.0162722	0.663683	
rs7616632	3	137031237	T	G	0.0132035	0.522135	Intelligence
rs2192528	4	18327896	A	G	0.0133687	0.480065	
rs17427571	4	82254908	A	G	0.0138255	0.684313	
rs35531607	4	92533225	T	C	-0.0128402	0.525917	
rs13109404	4	102896591	T	G	0.0312035	0.928024	
rs365663	5	1428883	A	G	0.0146291	0.545963	
rs460692	5	3126584	C	T	0.0210557	0.137484	
rs56372231	5	102321905	C	T	-0.0169439	0.665907	
rs180769	5	135615615	T	C	0.0127243	0.424698	
rs11567976	5	137654218	C	T	-0.0128042	0.429092	
rs151014368	5	176751059	G	A	-0.0160924	0.793742	
rs72838268	6	27021173	A	G	0.0167192	0.77265	
rs34556183	6	28584775	A	G	0.0169228	0.719606	
rs1633005	6	29764472	C	T	0.0174716	0.791845	Sarcoidosis
rs80193650	6	33464363	A	G	-0.0168389	0.837534	
rs113113059	6	43160375	T	C	0.0161406	0.78	
rs9382445	6	54937974	T	C	0.014536	0.62305	
rs2231265	6	89790201	A	G	-0.014955	0.227711	
rs9345234	6	93162639	A	C	-0.0130117	0.421984	
rs34731055	7	2106928	C	T	-0.0194603	0.81911	
rs2079070	7	114126432	C	G	0.0175475	0.264613	
rs7806045	7	132610266	T	C	0.0147916	0.754703	
rs330088	8	9149746	T	C	-0.0144687	0.452988	
rs4333549	8	10979561	C	G	0.0139369	0.47293	
rs73219758	8	14279446	G	A	0.0164012	0.708064	
rs10973207	9	37100525	G	T	-0.0204339	0.842323	
rs1776776	9	140497072	T	C	0.019963	0.873832	
rs12246842	10	21830580	A	G	0.0133949	0.459815	
rs10761674	10	64618340	C	T	0.0123329	0.477334	
rs11190970	10	103128332	G	A	0.015379	0.798661	
rs7915425	10	125016501	T	C	0.0190638	0.174682	Neutropenia, response to gemcitabine, pancreatic carcinoma
rs1517572	11	28829882	A	C	-0.0146443	0.419464	
rs4592416	11	43800474	A	G	-0.0146828	0.535593	
rs11039544	11	48173412	G	A	0.018389	0.837779	
rs174560	11	61581764	T	C	-0.0135751	0.685785	Chronic kidney disease, serum metabolite measurement
rs12791153	11	80685181	A	T	-0.0235481	0.918911	
rs1553132	11	88297740	A	G	-0.0145068	0.741567	

rs1939455	11	101520886	G	T	0.0204253	0.879446	
rs1079727	11	113289182	T	C	-0.0182922	0.842182	
rs7115462	11	113408517	G	A	-0.0265639	0.92647	
rs1263056	11	116576415	A	G	0.0127992	0.519099	
rs7951019	11	118358027	T	G	-0.0368792	0.967773	
rs3751046	11	122828342	A	G	-0.0194129	0.853507	
rs34354917	12	38764559	C	A	0.0137464	0.710472	
rs11614986	12	110007939	A	G	0.016379	0.820952	
rs4767550	12	117951150	A	G	-0.0143001	0.585862	
rs6575005	14	26954078	T	C	0.0155637	0.757854	
rs10483350	14	29816155	A	G	-0.017369	0.804582	
rs61985058	14	60233841	C	T	-0.0185938	0.856824	
rs55658675	14	65554638	C	T	0.0131415	0.644938	
rs11621908	14	78495761	C	T	0.0240951	0.917141	
rs8038326	15	47989799	A	G	0.0159204	0.72691	
rs3095508	16	6550400	C	A	0.0153518	0.593529	
rs11643715	16	23909538	C	G	-0.013895	0.709058	
rs9937053	16	53799507	G	A	0.0169348	0.576695	
rs8050478	16	56120461	G	A	0.0160009	0.500253	
rs7503199	17	8134275	C	T	0.0147449	0.734267	
rs205024	17	11227352	C	T	-0.0138261	0.616265	
rs8072993	17	21335627	T	G	-0.0174776	0.363492	
rs147114641	17	43581015	C	A	0.0159801	0.773722	
rs2696429	17	44335274	G	A	0.0168884	0.773626	
rs9895274	17	45539117	C	T	0.0133426	0.510408	
rs9903973	17	50571227	C	T	0.0127747	0.46702	
rs12607679	18	53059748	T	C	0.0201387	0.737717	
rs10421649	19	9942262	T	A	-0.0132975	0.44303	
rs2072727	20	43538733	T	C	0.0132425	0.43617	Chronotype measurement

Supplementary Table 3. Descriptive statistics for UK Biobank participants of European ancestry (N = 317,723) included in the main analyses of sleep duration and PhenoAge acceleration, according to self-reported sleep duration.

Characteristics	All	Short sleep, <6 h/d	Intermediate sleep, 6-8 h/d	Long sleep, >8 h/d
No. of participants	384,490	19,928	335,202	29,360
Sleep duration, h/d	7.16 (1.08)	4.76 (0.52)	7.12 (0.73)	9.30 (0.64)
Age at recruitment, y	56.8 (8.02)	57.2 (7.70)	56.6 (8.03)	58.8 (7.90)
Sex (women), n (%)	206642 (53.7%)	11162 (56.0%)	179092 (53.4%)	16388 (55.8%)
Education				
Degree, n (%)	315151 (82.7%)	13978 (71.2%)	279810 (84.2%)	21363 (73.6%)
No degree, n (%)	66008 (17.3%)	5651 (28.8%)	52683 (15.8%)	7674 (26.4%)
Body mass index, kg/m2	27.4 (4.75)	28.5 (5.45)	27.3 (4.66)	28.1 (5.09)
Smoking status				
Never, n (%)	206510 (53.9%)	9732 (49.1%)	182171 (54.5%)	14607 (50.0%)
Previous, n (%)	136638 (35.7%)	6996 (35.3%)	118480 (35.5%)	11162 (38.2%)
Current, n (%)	40062 (10.5%)	3094 (15.6%)	33501 (10.0%)	3467 (11.9%)
Drinking status				
Never, n (%)	12340 (3.21%)	1036 (5.21%)	10017 (2.99%)	1287 (4.39%)
Previous, n (%)	13271 (3.45%)	1290 (6.49%)	10479 (3.13%)	1502 (5.12%)
Current, n (%)	358575 (93.3%)	17560 (88.3%)	314470 (93.9%)	26545 (90.5%)
IPAQ activity group				
High, n (%)	126921 (40.5%)	6218 (41.0%)	111974 (40.8%)	8729 (37.2%)
Moderate, n (%)	127813 (40.8%)	5507 (36.3%)	112751 (41.1%)	9555 (40.8%)
Low, n (%)	58433 (18.7%)	3429 (22.6%)	49854 (18.2%)	5150 (22.0%)
Major diseases				
Cardiovascular disease, n (%)	22051 (5.74%)	1879 (9.45%)	17307 (5.17%)	2865 (9.78%)
Hypertension, n (%)	103404 (26.9%)	6797 (34.2%)	86839 (25.9%)	9768 (33.3%)
Diabetes mellitus, n (%)	18386 (4.79%)	1420 (7.16%)	14568 (4.35%)	2398 (8.19%)
Component of biological age				
Lymphocyte (%)	28.7 (7.34)	28.3 (7.54)	28.7 (7.31)	28.2 (7.56)
Mean cell volume (fL)	82.9 (5.25)	83.0 (5.52)	82.8 (5.20)	83.2 (5.59)
Serum glucose (mmol/L)	5.11 (1.21)	5.21 (1.42)	5.10 (1.16)	5.26 (1.51)
Red cell distribution width (%)	13.5 (0.95)	13.6 (1.08)	13.5 (0.93)	13.6 (1.04)
White blood cell count (1000 cells/uL)	6.89 (1.93)	7.14 (1.99)	6.85 (1.91)	7.13 (2.11)
Albumin (g/L)	45.2 (2.61)	45.1 (2.69)	45.3 (2.59)	45.0 (2.67)
Creatinine (umol/L)	72.2 (16.2)	71.7 (18.3)	72.2 (15.8)	73.3 (19.4)
C-reactive protein (mg/dL)	0.26 (0.44)	0.32 (0.50)	0.25 (0.42)	0.32 (0.51)
Alkaline phosphatase (U/L)	83.5 (26.1)	88.2 (30.1)	82.9 (25.6)	86.7 (28.8)
Biological ages, y				
PhenoAge	50.8 (9.42)	**52.0 (9.30)**	**50.4 (9.34)**	**53.7 (9.82)**
PhenoAge acceleration	0.00 (4.66)	**0.80 (5.39)**	**-0.12 (4.53)**	**0.87 (5.39)**

Baseline characteristics of UK Biobank participants were presented as mean values (standard deviation) for continuous variables and n (%) for categorical variables.

Supplementary Table 4. Descriptive statistics for UK Biobank participants of European ancestry (N = 317,723) included in the main analyses of sleep duration and BioAge acceleration, according to self-reported sleep duration.

Characteristics	All	Short sleep, <6 h/d	Intermediate sleep, 6-8 h/d	Long sleep, >8 h/d
No. of participants	317,723	15,821	278,440	23,462
Sleep duration, h/d	7.16 (1.06)	4.78 (0.51)	7.12 (0.73)	9.28 (0.62)
Age at recruitment, y	56.6 (8.04)	57.0 (7.76)	56.4 (8.04)	58.7 (7.97)
Sex (women), n (%)	171835 (54.1%)	8961 (56.6%)	149596 (53.7%)	13278 (56.6%)
Education				
Degree, n (%)	263167 (83.5%)	11368 (72.8%)	234480 (84.9%)	17319 (74.6%)
No degree, n (%)	51954 (16.5%)	4238 (27.2%)	41827 (15.1%)	5889 (25.4%)
Body mass index, kg/m2	27.3 (4.67)	28.4 (5.29)	27.2 (4.59)	27.9 (5.00)
Smoking status				
Never, n (%)	172605 (54.5%)	7923 (50.4%)	152827 (55.1%)	11855 (50.7%)
Previous, n (%)	112269 (35.5%)	5575 (35.4%)	97811 (35.2%)	8883 (38.0%)
Current, n (%)	31820 (10.0%)	2236 (14.2%)	26954 (9.71%)	2630 (11.3%)
Drinking status				
Never, n (%)	10002 (3.15%)	788 (4.99%)	8216 (2.95%)	998 (4.26%)
Previous, n (%)	10414 (3.28%)	956 (6.06%)	8336 (3.00%)	1122 (4.79%)
Current, n (%)	297067 (93.6%)	14040 (89.0%)	261702 (94.1%)	21325 (91.0%)
IPAQ activity group				
High, n (%)	106018 (40.9%)	5080 (41.9%)	93744 (41.0%)	7194 (38.3%)
Moderate, n (%)	105833 (40.8%)	4414 (36.4%)	93756 (41.0%)	7663 (40.8%)
Low, n (%)	47654 (18.4%)	2623 (21.6%)	41091 (18.0%)	3940 (21.0%)
Major diseases				
Cardiovascular disease, n (%)	16096 (5.07%)	1260 (7.98%)	12804 (4.60%)	2032 (8.68%)
Hypertension, n (%)	82698 (26.1%)	5178 (32.8%)	69991 (25.2%)	7529 (32.2%)
Diabetes mellitus, n (%)	14409 (4.54%)	1044 (6.63%)	11570 (4.16%)	1795 (7.67%)
Component of biological age				
FEV_1 (L)	2.85 (0.80)	2.71 (0.81)	2.87 (0.80)	2.68 (0.75)
SBP (mm Hg)	138 (18.5)	138 (18.3)	138 (18.4)	139 (19.0)
Total Cholesterol (mg/dL)	221 (43.9)	222 (45.5)	221 (43.5)	220 (47.1)
Glycated hemoglobin (%)	3.58 (0.63)	3.67 (0.72)	3.57 (0.61)	3.67 (0.76)
Blood urea nitrogen (mg/dL)	15.2 (3.82)	15.2 (4.10)	15.1 (3.76)	15.5 (4.27)
Albumin (g/L)	45.3 (2.59)	45.2 (2.66)	45.3 (2.58)	45.1 (2.65)
Creatinine (umol/L)	72.2 (17.1)	71.5 (18.8)	72.2 (16.4)	73.3 (23.7)
C-reactive protein (mg/dL)	0.25 (0.41)	0.30 (0.46)	0.24 (0.40)	0.30 (0.48)
Alkaline phosphatase (U/L)	83.0 (25.9)	87.4 (29.5)	82.5 (25.4)	85.9 (28.3)
Biological ages, y				
BioAge	53.9 (8.67)	**54.9 (8.36)**	**53.7 (8.65)**	**56.5 (8.59)**
BioAge acceleration	0.00 (3.31)	**0.61 (3.47)**	**-0.08 (3.28)**	**0.49 (3.49)**

Baseline characteristics of UK Biobank participants were presented as mean values (standard deviation) for continuous variables and n (%) for categorical variables.

Supplementary Table 5. Descriptive statistics for UK Biobank participants of European ancestry (N = 442,664) included in the main analyses of sleep duration and telomere length, according to self-reported sleep duration.

Characteristics	All	Short sleep, <6 h/d	Intermediate sleep, 6-8 h/d	Long sleep, >8 h/d
No. of participants	442,664	23,030	385,786	33,848
Sleep duration, h/d	7.16 (1.08)	4.76 (0.52)	7.12 (0.73)	9.30 (0.64)
Age at recruitment, y	56.8 (8.03)	57.2 (7.71)	56.6 (8.03)	58.8 (7.90)
Sex (women), n (%)	239936 (54.2%)	12977 (56.3%)	207921 (53.9%)	19038 (56.2%)
Education				
Degree, n (%)	363075 (82.7%)	16186 (71.3%)	322253 (84.2%)	24636 (73.6%)
No degree, n (%)	75785 (17.3%)	6501 (28.7%)	60436 (15.8%)	8848 (26.4%)
Body mass index, kg/m2	27.4 (4.76)	28.5 (5.47)	27.3 (4.67)	28.1 (5.13)
Smoking status				
Never, n (%)	237954 (53.9%)	11218 (49.0%)	209880 (54.6%)	16856 (50.0%)
Previous, n (%)	157078 (35.6%)	8075 (35.3%)	136152 (35.4%)	12851 (38.1%)
Current, n (%)	46137 (10.5%)	3611 (15.8%)	38519 (10.0%)	4007 (11.9%)
Drinking status				
Never, n (%)	14243 (3.22%)	1204 (5.24%)	11541 (2.99%)	1498 (4.43%)
Previous, n (%)	15299 (3.46%)	1496 (6.51%)	12062 (3.13%)	1741 (5.15%)
Current, n (%)	412778 (93.3%)	20280 (88.3%)	361920 (93.9%)	30578 (90.4%)
IPAQ activity group				
High, n (%)	146179 (40.5%)	7155 (40.9%)	128972 (40.8%)	10052 (37.3%)
Moderate, n (%)	147130 (40.8%)	6340 (36.3%)	129825 (41.1%)	10965 (40.7%)
Low, n (%)	67260 (18.7%)	3989 (22.8%)	57324 (18.1%)	5947 (22.1%)
Major diseases				
Cardiovascular disease, n (%)	25440 (5.76%)	2207 (9.61%)	19903 (5.17%)	3330 (9.86%)
Hypertension, n (%)	118845 (26.9%)	7889 (34.3%)	99691 (25.9%)	11265 (33.4%)
Diabetes mellitus, n (%)	21267 (4.81%)	1667 (7.27%)	16815 (4.37%)	2785 (8.25%)
White blood cell (leukocyte) count, 10^9 cells/Litre	6.90 (2.04)	7.15 (1.98)	6.86 (2.03)	7.14 (2.21)
Z-standardized leucocyte telomere length values	-0.01 (0.99)	-0.07 (0.98)	-0.01 (0.99)	-0.09 (1.01)

Baseline characteristics of UK Biobank participants were presented as mean values (standard deviation) for continuous variables and n (%) for categorical variables.
